# Toll-Like Receptor 4 Mediated Oxidized Low-Density Lipoprotein-Induced Foam Cell Formation in Vascular Smooth Muscle Cells via Src and Sirt1/3 Pathway

**DOI:** 10.1155/2021/6639252

**Published:** 2021-04-09

**Authors:** Zhongli Chen, Qiqi Xue, Lijuan Cao, Yanpin Wang, Yuanyuan Chen, Xiaojie Zhang, Fan Xiao, Ying Yang, Melvin R. Hayden, Yan Liu, Ke Yang

**Affiliations:** ^1^Department of Vascular & Cardiology, Ruijin Hospital, Shanghai Jiaotong University School of Medicine, Shanghai 200025, China; ^2^Department of Geratology, Ruijin Hospital, Shanghai Jiaotong University School of Medicine, Shanghai 200025, China; ^3^Department of Cardiology, Huangpu Branch, Shanghai Ninth People's Hospital, Shanghai Jiaotong University School of Medicine, Shanghai 200011, China; ^4^Department of Electrocardiogram, Zhongshan Hospital, Fudan University, Shanghai 200032, China; ^5^Department of Cardiology, Shanghai Ninth People's Hospital, Shanghai Jiaotong University School of Medicine, Shanghai 200011, China; ^6^Department of Endocrinology, The Second People's Hospital of Yunnan Province, Kunming, Yunnan 650021, China; ^7^Departments of Internal Medicine, Endocrinology Diabetes and Metabolism, Diabetes and Cardiovascular Disease Center, University of Missouri-Columbia School of Medicine, Columbia, 65201 Missouri, USA

## Abstract

Oxidized low-density lipoprotein (oxLDL) induced a foam-cell-like phenotype of the vascular smooth muscle cells (VSMCs), leading to the inflammatory responses incorporating Toll-like receptor- (Tlr-) mediated cellular alterations. However, the role of Tlr4 in foam cell formation and underlying molecular pathways has not been comprehensively elucidated. To further investigate the mechanism, VSMCs were incubated with different doses of oxLDL, and then, the lipid, reactive oxygen species (ROS) accumulation, Tlr family genes, and the foam cell phenotype were explored. We observed that oxLDL induced foam cell-like phenotype in VSMCs and led to lipid and ROS accumulation in a dose-dependent manner. Furthermore, in the Tlr family, Tlr4 demonstrated the strongest upregulation under oxLDL stimulation. Simultaneously, oxLDL induced activation of Src, higher expression of Nox2, and lower expression of Mnsod, Sirt1, and Sirt3. By interfering the TLR4 expression, the phenotype alteration, lipid accumulation in VSMCs, and Src kinase activation induced by oxLDL were abolished. After interfering Src activation, the oxLDL-induced lipid accumulation and foam cell phenotype in VSMCs were also alleviated. Furthermore, the ROS accumulation, upregulated Nox2 expression, downregulated Sirt1, Sirt3, and Mnsod expression in VSMCs under oxLDL stimulation were also relieved after the knockdown of Tlr4. Additionally, overexpression of Sirt1 and Sirt3 ameliorated the ROS accumulation and foam cell-like marker expression in VSMCs. These results demonstrated that beyond its familiar role in regulating inflammation response, Tlr4 is a critical regulator in oxLDL-induced foam cell formation in VSMCs via regulating Src kinase activation as well as Sirt1 and Sirt3 expression.

## 1. Introduction

Coronary artery disease (CAD) is a leading health burden contributing to high morbidity and mortality worldwide [1]. And atherosclerosis serves as the major cause of driving the occlusion of coronary arteries and cardiovascular events [2]. During the process of atherosclerosis, mounting foam cell formation and necrosis invoked the inflammation storm, which aggravated instability of plaque and led to acute myocardial infarction [3, 4]. Previous studies showed that despite the well-established essential role of monocyte-derived macrophages, vascular smooth muscle cells (VSMCs) were equipped with macrophage features, constituting a substantial source of foam cells and inflammatory response in plaques [5, 6]. Basically, the oxidized low-density lipoprotein (oxLDL) can be ingested by VSMCs [7]. As a result, the VSMC underwent phenotype transition from the classical contractile state to the macrophage-like phenotype [8]. Such VSMC-derived foam cells accelerated the progression of atherosclerosis [9, 10]. Though a few scavenger receptors participated in lipid uptake during foam cell formation, the specific mechanism contributing to lipid accumulation in VSMCs was still unclear. To acquire a better understanding of VSMC alteration in atherosclerosis, it is necessary to clarify the mechanism underlying the lipid accumulation in VSMCs.

Along with the continuous formation and necrosis of the foam cells, regional inflammatory storms induced by excessive cytokines caused damage to the vessels [11]. oxLDL stimulation induced foam cell formation and accelerated mitochondrial oxidative stress [12, 13], which led to accumulating reactive oxygen species (ROS) production [13]. Taken together, these factors evoked inflammatory response signalling pathways in foam cells [14, 15]. In our previous studies, oxLDL activated the proinflammatory signalling pathway and raised the expression and secretion of inflammatory cytokines in VSMCs via Tlr4 [16]. Moreover, oxLDL promoted the bond of Tlr4 with Src kinase to induce lipid uptake and foam cell formation in macrophages [17]. These results hint that Tlr4 might also regulate the lipid uptake process and contribute to foam cell formation in VSMCs. Nevertheless, such a potential role of Tlr4 in the foam cell formation in VSMCs has not been comprehensively elaborated.

Additionally, excessive production of reactive oxygen species (ROS) was widely observed in atherosclerosis. Although it has been well-established that the broken oxidative homeostasis could promote the vascular inflammation response, the relationship between oxidative stress and foam cell formation in VSMCs has not been elucidated. Although a previous study showed that Tlr4 mediated ROS accumulation via regulating Nox2 [18], other mechanisms underlying the ROS accumulation process in VSMCs were still limited. Currently, the pivotal roles of the emerging sirtuin family in maintaining the balance of ROS metabolism arouse increasing attention [19]. However, whether they were involved in oxLDL-TLR4 induced VSMC ROS accumulation remains obscure.

In this study, we hypothesized that Tlr4 mediated oxLDL-induced foam cell formation via regulating lipid accumulation and ROS production in VSMCs. Based on cellular and molecular research, we aimed to clarify the mechanism underlying the ROS and lipid accumulation induced by oxLDL in VSMCs, thus deepening the insights about the formation of foam cell-like VSMCs during atherosclerosis.

## 2. Methods

### 2.1. Reagents and Antibodies

Fetal bovine serum (Cat#16000), advanced DMEM/F-12 (Cat#12634010), and antibiotic-antimycotic (Cat#15240096) were obtained from Gibco (CA, USA). oxLDL (Cat#L34357), Nile Red (Cat#N1142), Image-IT™ LIVE Green Reactive Oxygen Species Detection Kit (Cat#I36007), MitoSOX™ Red Mitochondrial Superoxide Indicator (Cat#M36008), DAPI (Cat#D1306), TRIzol™ Reagent (Cat#15596026), Goat anti-Rabbit secondary antibody conjunction with Alexa Fluor Plus 555 (Cat#A-21429), Goat anti-Mouse secondary antibody conjunction with Alexa Fluor Plus 488 (Cat#A-11001), and Lipofectamine™ RNAi MAX transfection reagent (Cat#13778150) were purchased from Invitrogen (CA, USA). Tlr4 siRNA (Cat#sc-40261), Src siRNA (Cat#sc-29859), and negative control siRNA (Cat#sc-37007) were bought from Santa Cruz (TX, USA). Let-blank (Cat#GCNL), Let-Sirt1 (+) (Cat#GCD0161581), and Let-sirt3 (+) (Cat#GCD0201555) were acquired from Shanghai Genechem (Shanghai, China). Premix ex taq™ DNA polymerase (Cat#RR039A) and primeScript™ 1st strand cDNA synthesis kit (Cat#6110A) were collected from TaKaRa (Tokyo, Japan). HDL and LDL/VLDL cholesterol assay kit (Cat#ab65390), H&E staining kit (Cat#ab245880), BCA protein assay kit (Cat#ab102536), PP2 (Cat#ab120308), PP3 (Cat#ab120617), and the primary antibody of Myh11 (Cat#ab53219), *α*Sma (Cat#ab52218), Mac2 (Cat#ab2785), Cd68 (Cat#ab31630), Nox2 (Cat#ab80508), Nox4 (Cat#ab14544), and Sirt4 (Cat#ab124521) were purchased from Abcam (MA, USA). Additionally, primary antibodies of *β*-actin (Cat#3700), p-Src Y418 (Cat#6943), t-Src (Cat#2123), Mnsod (Cat#13141), Sirt1 (Cat#2314), Sirt2 (Cat#12650), Sirt3 (Cat#5490), Sirt5 (Cat#8782), Sirt6 (Cat#12486), Sirt7 (Cat#5360), and tlr4 (Cat#14358) as well as the second antibody-conjunction HRP anti-mouse/rabbit (Cat#7076/Cat#7074) were gained from CST (MA, USA).

### 2.2. Animals

All animal experiments were conducted according to the Guide for the Care and Use of Laboratory Animals and approved by the Animal Care and Use Committee of Shanghai Jiao Tong University, China, which conform to the guidelines from Directive 2010/63/EU of the European Parliament on the protection of animals used for scientific purposes. Before obtaining the aortic tissues, the mice were firstly anesthetized and were euthanized humanely by intraperitoneal injection of sodium pentobarbital.

### 2.3. Primary Smooth Muscle Cell Culture

Wild-type (C57BL/6) mice were purchased from the Model Animal Research Center of Nanjing University (Nanjing, China) and were euthanized at 4 weeks old. To obtain the primary smooth muscle cells, the aortas of the mice were dissected, and the adventitia was removed. The aortic explants were cultured after mechanical dissection and twice washing in PBS. The explant-derived VSMCs were cultured at 37°C, 5% CO_2_ in F12 : DMEM (1 : 1) medium with 20% fetal bovine serum and 1% antibiotic-antimycotic.

### 2.4. Assessment of Intracellular Lipids

VSMCs were cultured in 6-well plates and incubated with oxLDL for 72 hours. Afterwards, the VSMCs were washed by PBS twice and then were fixed in 4% paraformaldehyde/PBS for 15 minutes. Then, the cells were stained by 100 ng/mL Nile Red for intracellular lipid detection [20]. All cell samples were observed and photographed microscopically (ZEISS LSM 800, Zeiss Microsystems). Five fields of view were randomly acquired, and representative images were shown. Intracellular lipids were quantified by HDL and LDL/VLDL Cholesterol Assay Kit. Quantifications of total lipoprotein were conducted according to the description in the manufacturer's protocols and demonstrated as relative value-to-total protein ratio (*n* = 3).

### 2.5. Intracellular Reactive Oxygen Species Assay

Cellular ROS was detected by the carboxy-H2DCFDA kit. Based on the kit instruction, a 25 *μ*M carboxy-H2DCFDA working solution was used to label VSMCs for 30 minutes at 37°C in the dark. Afterwards, cells were gently washed in warm HBSS/Ca/Mg and incubated with Hoechst 33342 for another 5 minutes. After three times washes, the ROS signals were observed via a fluorescent microscope. Five fields of view were randomly acquired, and representative images were shown.

### 2.6. Assessment of Intracellular Mitochondrial Superoxide

After incubation with oxLDL, VSMCs were gently washed by warm Hanks buffer. The mitochondrial superoxide was detected using the MitoSOX™ Red indicator. According to the manufacturer's recommendation, the MitoSOX™ reagent was diluted into a working concentration and added to the six-well plates covering the VSMCs. After a 10-minute incubation in MitoSOX™ reagent at 37°C in the dark, the cells were washed by Hanks buffer three times. Images were obtained by a fluorescence microscope using a green excitation light. Five fields of view were randomly acquired, and representative images were shown.

### 2.7. Quantitative Real-Time Polymerase Chain Reactions

Total RNA was extracted by TRIzol reagent, and five *μ*gs of total RNA undergone the reverse-transcription. Polymerase chain reactions (PCR) were carried out using Power SYBR Green PCR Master Mix (Applied Biosystems, CA, USA) according to the manufacturer's recommendation in a StepOne System (Applied Biosystems, CA, USA). Primers for the promoter sequences are listed in Table 1. Gene expression was normalized with *β*-actin as the reference gene. The StepOne software v2.1 (Applied Biosystems) was used for data analysis.

### 2.8. Western Blot

VSMCs were lysed in Western & IP Cell lysate on ice for 15 min. The total protein was collected after centrifugation. Protein concentration was measured using a BCA-protein assay kit. Equal quantification of protein (20 *μ*g) was applied in a 15% SDS-polyacrylamide gel and transferred to polyvinylidene fluoride (PVDF) membranes. The membranes were blocked by 5% milk for 1 hour at room temperature and then incubated with the primary antibody overnight at 4°C. After being washed three times in TBS buffer, the membrane was incubated with horseradish peroxidase- (HRP-) conjugated secondary antibodies at room temperature for 2 hours. Finally, images were captured in a Tanon-5500 chemiluminescent imaging system (Tanon Science and Technology Co., Ltd., Shanghai, China) and quantified by ImageJ software (Bio-Rad, Hercules, CA, USA).

### 2.9. Oligonucleotide Transfection

RNA interference was conducted using the Oligofectamine reagent (Invitrogen). And cultured VSMCs were transfected with the targeting siRNA and negative control siRNA (non-targeting sequence) according to the instruction. Cells had 60%-70% confluency on the day of transfection. After transfecting for 48 hours, the knockdown efficiency was tested by western blot.

### 2.10. Pretreatment with PP2 and PP3

PP2 (10 *μ*mol/L) and PP3 (10 *μ*mol/L) were, respectively, added to VSMCs and incubated for 30 min before oxLDL stimulation. PP3 served as the negative control for PP2.

### 2.11. Sirt1 and Sirt3 Overexpression Lentivirus Production and Transfection

The Sirt1 and Sirt3 overexpression was completed using recombinant lentivirus vectors containing the overexpression plasmid of the corresponding gene. Empty vector lentivirus was also transfected as control. Cells were infected with lentivirus for 72 hours, followed by an RT-PCR for efficiency determination.

### 2.12. Statistical Analysis

Values were showed as mean with standard deviation (SD). Paired samples were compared using Student's paired *t*-test. One-way ANOVA followed by Friedman's post test or two-way ANOVA followed by the Dunnett multiple comparison procedure was also used to determine significance as appropriate. A two-sided *p* value less than 0.05 was considered statistically significant. Data were analyzed and plotted using the Graphpad Prism Version 7.0.

## 3. Results

### 3.1. oxLDL Induced Dose-Dependent Lipid Accumulation, Oxidative Stress, and Foam Cell Formation in Vascular Smooth Muscle Cells

**Figure 1:**
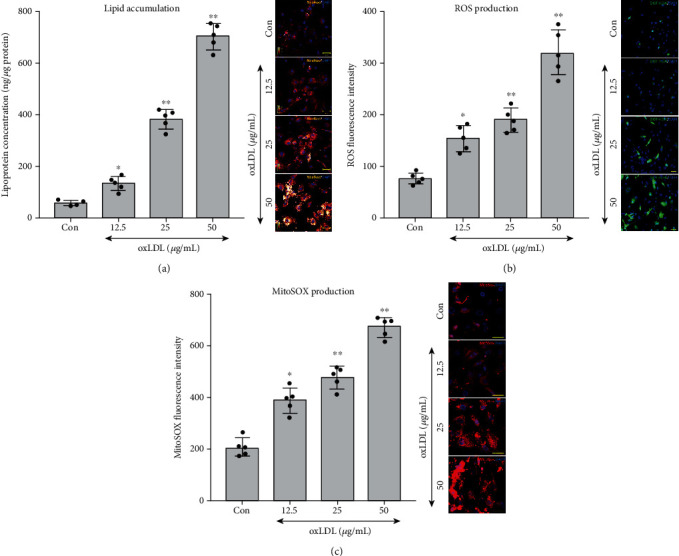
Oxidized low-density lipoprotein (oxLDL) induced lipid accumulation, reactive oxygen species (ROS), and mitochondrial superoxide production in vascular smooth muscle cells (VSMCs). After treating with doses of oxLDL (12.5, 25, and 50 *μ*g) for 48 hours, the lipid accumulation, ROS, and mitochondrial superoxide in VSMCs were measured, with the untreated group serving as a control reference (Con). (a) Nile Red (orange) was used to stain the lipid, and the concentration of lipoprotein was measured. (b) DCFH-DA-labelled (green) ROS and density of ROS production were detected. (c) MitoSOX-labelled (red) mitochondrial superoxide and level of superoxide were calculated. (*n* = 5 per group, results were expressed as mean ± SD; ^∗^*P* < 0.05 and ^∗∗^*P* < 0.01, as compared with the Con group).

After being stimulated by gradient dose (12.5, 25, and 50 *μ*g/mL) of oxLDL for 48 hours, lipid accumulation, cellular ROS accumulation, mitochondrial superoxide generation, and foam cell formation were examined. The Nile Red staining and lipoprotein quantification showed that oxLDL induced lipid accumulation in VSMCs in a dose-dependent manner (Figure 1(a)). The DCFH-DA and MitoSOX served as detectors for labelling cellular ROS and mitochondrial superoxide generation, respectively. Similar to the oxLDL-induced lipid accumulation, a higher concentration of oxLDL triggered severe oxidative stress within the cell and mitochondrial (Figures 1(b) and 1(c)).

**Figure 2:**
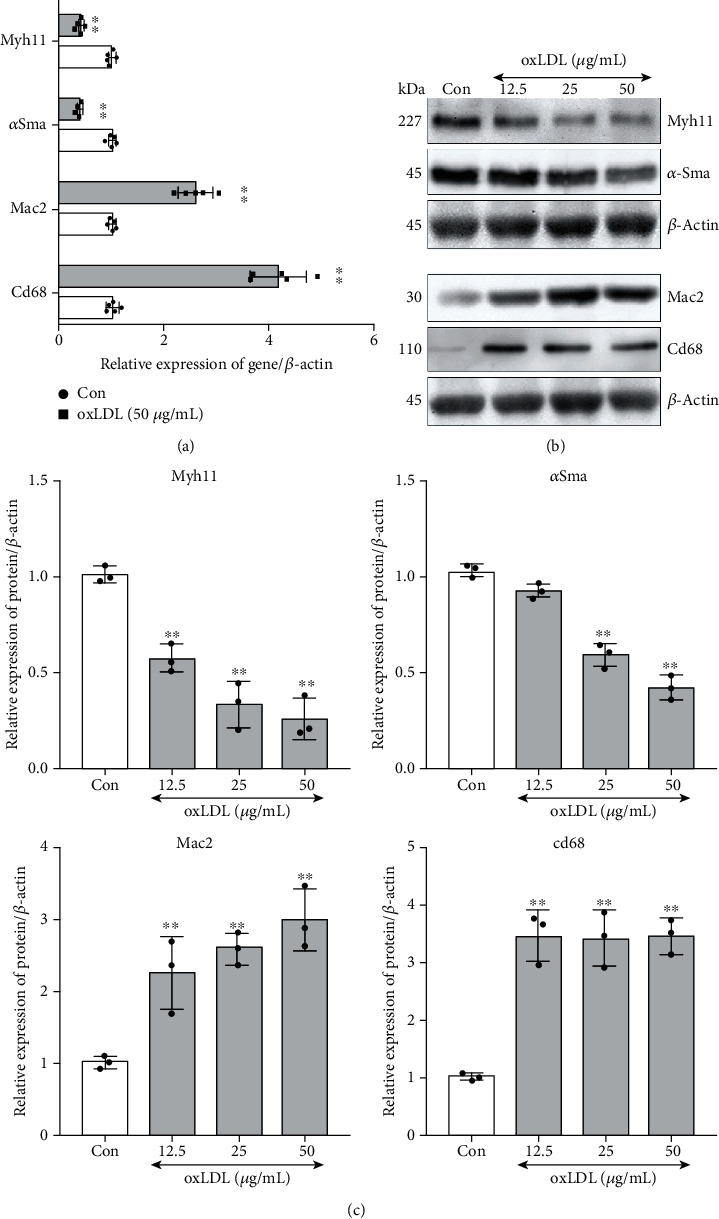
oxLDL promoted foam cell-like phenotype in VSMCs. After oxLDL stimulation, the mRNA or protein expression of genes (Myh11 and *α*Sma as VSMC contractile phenotype marker, Mac2, and Cd68 as foam cell marker) was detected by real-time PCR or western blot in VSMCs. The untreated group served as control (Con), and *β*-actin served as an internal reference gene to normalize protein expression. All of the real-time PCR results were calculated with 2^-*ΔΔ*CT^ method; the western blot results were calculated using grayscale value. (a) mRNA levels of *Myh11*, *αSma*, *Mac2*, and *Cd68* were measured by real-time PCR. (*n* = 5 per group, results were expressed as mean ± SD; (c) ^∗^*P* < 0.01, as compared with the Con group). (b, c) Protein expressions of Myh11, *α*Sma, Mac2, and Cd68 were measured by western blot (*n* = 3 per group, results were expressed as mean ± SD; ^∗∗^*P* < 0.01 as compared with the Con group).

Moreover, after oxLDL stimulation, the expression of *α*Sma and Myh11, the contractile phenotype-specific mRNA and protein, was downregulated, while the foam cells' phenotype markers, Cd68 and Mac2, were significantly upregulated (Figures 2(a)–2(c)).

### 3.2. oxLDL Mediated Significant Upregulation of Tlr4 along with Expression/Activation of Lipid Metabolism and Oxidative Stress Regulators

**Figure 3:**
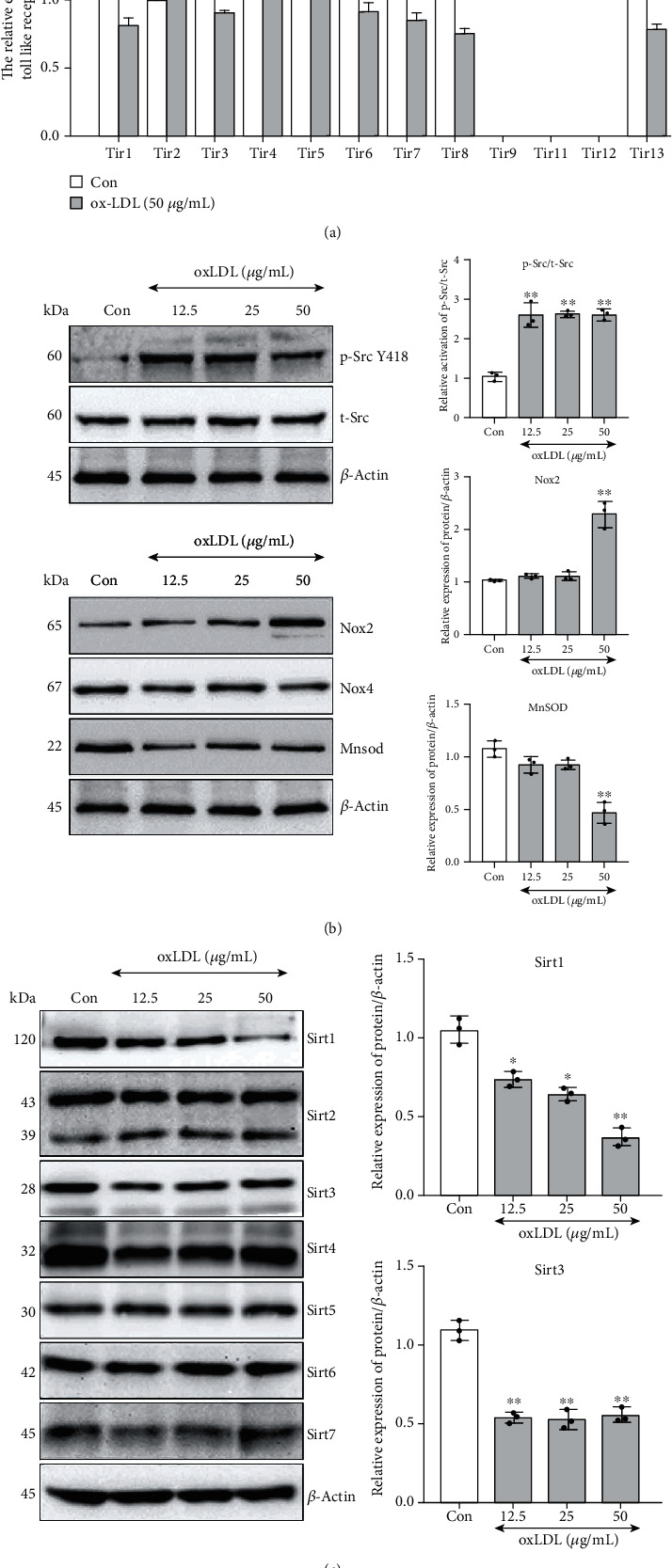
oxLDL regulated expression of toll-like receptor family, ROS-related genes, and sirtuin family as well as activation of Src. After oxLDL stimulation, the mRNA or protein expression of genes was detected by real-time PCR or western blot in VSMCs. The phosphorylation of Src was detected using western blot. The untreated group served as control (Con), and *β*-actin served as an internal reference gene to normalize protein expression. All of the real-time PCR results were calculated by 2^-*ΔΔ*CT^ method; the western blot results were calculated using grayscale value. (a) The mRNA expression of the toll-like receptor family ranging from *Tlr1* to *Tlr13*. (*n* = 3 per group, results were expressed as mean ± SD; ^∗^*P* < 0.01, as compared with the Con group.) (b) Upper: the phosphorylation of Src (Tyr-418, Y418) was detected after 1-hour stimulation by different doses (12.5, 25, and 50 *μ*g) of oxLDL in VSMCs. Bottom: the protein expression of ROS generation (Nox2 and Nox4) and elimination-related (Mnsod) genes in VSMCs, after stimulating by different doses (12.5, 25, and 50 *μ*g) of oxLDL for 48 hours. (c) After incubation with different doses (12.5, 25, and 50 *μ*g) of oxLDL, the protein levels of the sirtuin family (Sirt1-7) were measured. (*n* = 3 per group, results were expressed as mean ± SD; ^∗∗^*P* < 0.01 as compared with the Con group).

To explore the overall change of the Tlr family under the oxLDL stimulation in VSMCs, we further measured mRNA levels of the Tlrs in VSMCs after 48 hours of incubation with 50 *μ*g/mL of oxLDL to figure out which members of the Tlrs experienced a drastic change. Remarkably, under oxLDL treatment, expression of Tlr4 increased more significantly than any other Tlrs among Tlr1-Tlr13 (Figure 3(a)), with over 1.5-fold expression than the control. Since we previously observed the TLR4-Src participated in lipid accumulation in macrophages under oxLDL stimulation [17], we wonder whether the Src also changes in VSMCs after gradient dose of oxLDL stimulation. In line with the macrophage, we found that the phosphorylation site of Src (418-Tyr) was obviously activated in VSMCs. However, unlike macrophages, activation of Src in VSMCs did not show a dose-dependent effect, and the extent of activation was comparable across different oxLDL concentration-treated groups (upper of Figure 3(b)). Meanwhile, the expression of the ROS elimination-relevant gene Mnsod as well as Nox2 and Nox4, which are responsible for ROS generation, was examined. Only the highest dose (50 *μ*g/mL) of oxLDL resulted in significant elevation of Nox2 and remarkable decreased Mnsod, whereas the significant change of Nox4 expression was not observed (bottom of Figure 3(b)). Finally, the alteration of the oxidative balance maintainer, the sirtuin family, was also explored. It is interesting to note that only expression of Sirt1 and Sirt3 was remarkably downregulated while no significant effect was observed in terms of other members of the sirtuin family (Figure 3(c)).

### 3.3. Tlr4 Mediated oxLDL-Induced Lipid Accumulation, Oxidative Stress, and Foam Cell Formation in VSMCs

**Figure 4:**
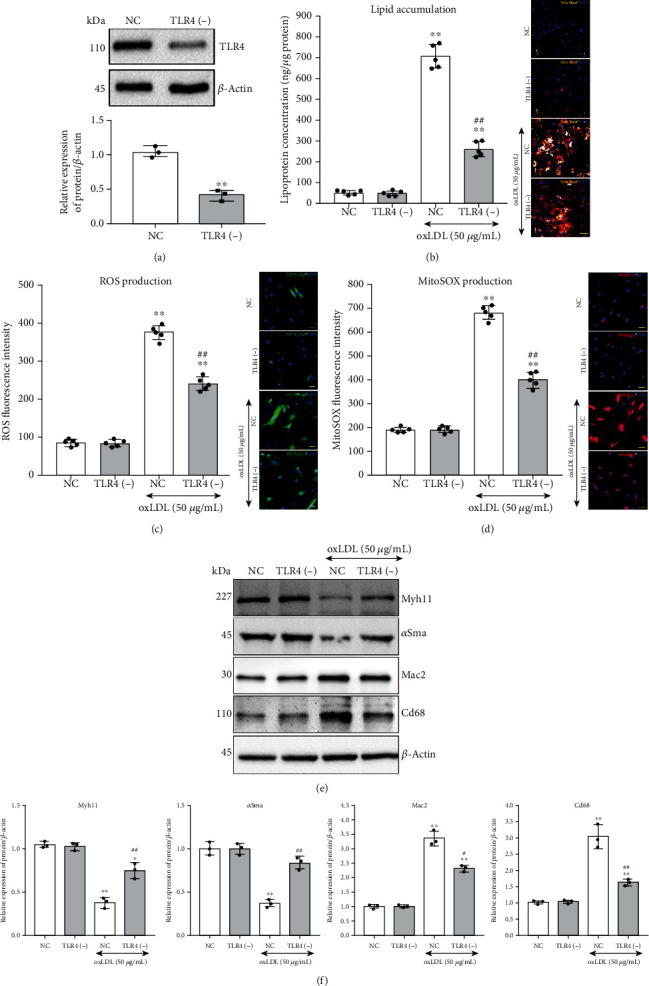
Knockdown of Tlr4 suppressed oxLDL-induced lipid accumulation, ROS and mitochondrial superoxide production, and regulated related gene activation and expression. Tlr4 was knockdown by siRNA transfection within VSMCs. (a) The Tlr4-specific [Tlr4(-)] and negative (NC) siRNA were transfected into VSMCs for 72 hours, and the knockdown efficiency was detected. (*n* = 3 per group, results were expressed as mean ± SD; compared with the NC group by Student's *t*-test, ^∗∗^*P* < 0.01.) Tlr4(-) or NC VSMCs were treated with or without oxLDL (50 *μ*g/mL) for 48 hours. (b) Lipid accumulation (Nile red stain, orange). (c) ROS (DCFH-DA, green) and (d) mitochondrial superoxide (MitoSOX, red) were measured. (*n* = 5 per group, results are expressed as mean ± SD; ^∗^*P* < 0.05, ^∗∗^*P* < 0.01, as compared with oxLDL-untreated NC group; ^#^*P* < 0.05, ^##^*P* < 0.01, as compared with oxLDL-treated NC group.) (e) Tlr4(-) or NC VSMCs were treated with or without oxLDL (50 *μ*g/mL) for 48 hours; western blot was conducted to measure oxidative stress-associated genes, VSMC contractile phenotype markers (Myh11 and *α*Sma), and foam cell markers (Mac2 and Cd68). And *β*-actin served as an internal reference gene to normalize protein expression. (f) All the western blot results were calculated using grayscale value. (*n* = 3 per group, results are expressed as mean ± SD; ^∗^*P* < 0.05, ^∗∗^*P* < 0.01, as compared with oxLDL-untreated NC group; ^#^*P* < 0.05, ^##^*P* < 0.01, as compared with oxLDL-treated NC group).

To investigate the role of Tlr4 in oxLDL-induced pathophysiological change and its relation with those altered regulators in VSMCs, Tlr4 was knockdown to further elaborate subsequent cellular phenomenon and molecular pathway. Tlr4 in VSMCs was significantly knocked down by targeted siRNA, compared with the negative control siRNA (NC) (Figure 4(a)). After 50 *μ*g/mL of oxLDL stimulation for 48 hours, lipid accumulation, ROS, and mitochondrial superoxide were still sharply promoted in the NC group. By contrast, these alterations were ameliorated in Tlr4-knockdown VSMCs (Figures 4(b)–4(d)). More importantly, although oxLDL still led to a significant decrease of VSMC contractile phenotype markers (Myh11 and *α*Sma) and elevated foam cell markers (Mac2 and Cd68) in NC VSMCs, TLR4 knockdown had interrupted most of these alterations, indicating that TLR4, at least partly, mediated the oxLDL-induced lipid and ROS accumulation and contributed to foam cell formation (Figures 4(e) and 4(f)).

### 3.4. Tlr4-Src Kinase Regulated Lipid Accumulation and Cellular Phenotype Transition in VSMCs

**Figure 5:**
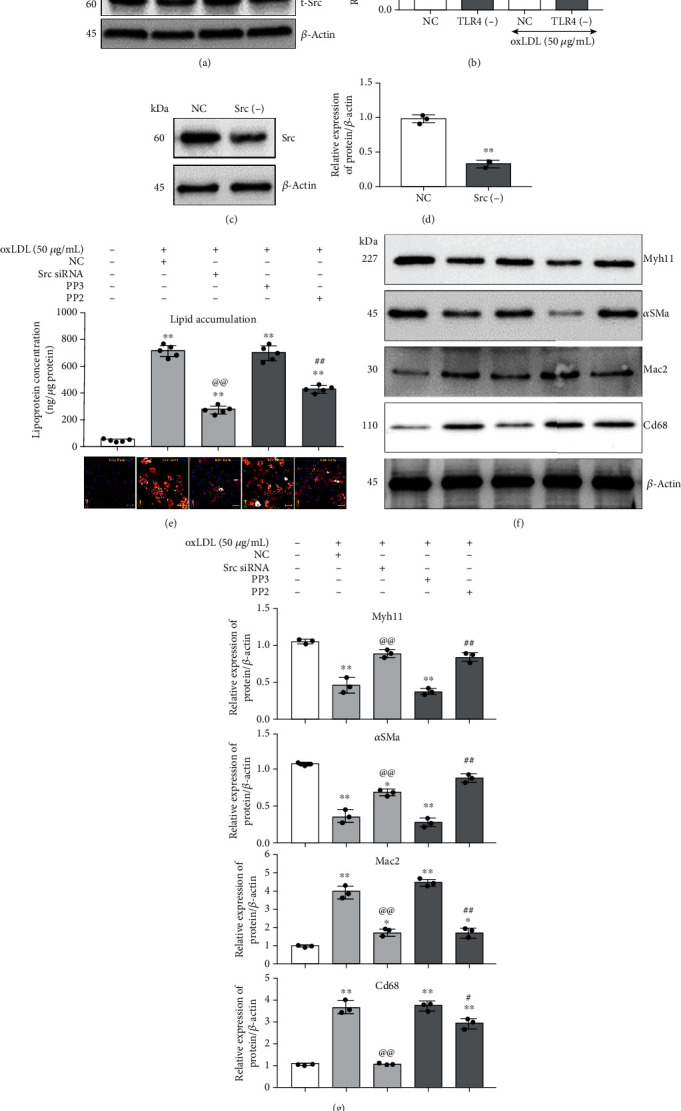
Knockdown or inactivation of Src regulated lipid accumulation and cellular phenotype in VSMCs. (a, b) Tlr4(-) or NC were treated with or without oxLDL (50 *μ*g/mL) for 1 hour; the activation of Src (Tyr-418, Y418) had been detected by western blot. (*n* = 3 per group, results were expressed as mean ± SD; ^∗∗^*P* < 0.01, as compared with oxLDL-untreated NC group; ^##^*P* < 0.01, as compared with oxLDL-treated NC group.) Src was knockdown by siRNA transfection within VSMCs. The Src-specific antagonist, PP2, was used to block Src activation, with PP3 serving as the negative control. The VSMCs were treated with and without 50 *μ*g/mL oxLDL for 48 hours. (c, d) The Src-specific [Src (-)] and negative control (NC) siRNA were, respectively, transfected into VSMCs for 72 hours, and the knockdown efficiency was detected. (*n* = 3 per group, results were expressed as mean ± SD; ^∗∗^*P* < 0.01, as compared with the NC group.) (e) Nile Red (orange) was used to label the lipid, and the concentration of intracellular lipid was measured. (*n* = 5 per group, results were expressed as mean ± SD; ^∗∗^*P* < 0.01, as compared with the untreated group; ^@@^*P* < 0.01, the oxLDL-treated Src siRNA group compared with the oxLDL-treated NC group; ^##^*P* < 0.01, the oxLDL-treated PP2 group compared with the oxLDL-treated PP3 group.) (f, g) Western blot was conducted to measure the VSMC contractile phenotype markers (Myh11 and *α*Sma) and foam cell markers (Mac2 and Cd68). All the western blot results were calculated using grayscale value, and *β*-actin served as an internal reference gene to normalize protein expression. (*n* = 3 per group, results were expressed as mean ± SD; ^∗∗^*P* < 0.01, as compared with the control group (untreated group); ^@@^*P* < 0.01, the oxLDL-treated Src siRNA group compared with the oxLDL-treated NC group; ^##^*P* < 0.01, the oxLDL-treated PP2 group compared with oxLDL-treated PP3 group).

Notably, after knocking down the Tlr4 in VSMCs, the activation in the Tyr-418 phosphorylation site of Src kinase was deprived to a great extent, compared with the NC group, after 1-hour oxLDL treatment (Figures 5(a) and 5(b)). Such an effect indicated that Tlr4 might regulate Src kinase activation under oxLDL stimulation. Moreover, we further hypothesized that Src kinase might be a downstream executor of TLR4 to impact lipid uptake in VSMCs. To illuminate this hypothesis, either the expression or the activation of Src was disturbed by siRNA or PP2, respectively, and then, the change of intracellular lipid concentration and cell phenotype markers was determined following oxLDL treatment. The efficiency of siRNA knockdown Src was tested by western blot (Figures 5(c) and 5(d)). Compared with the untreated group, higher intercellular lipid levels were observed in oxLDL-treated groups, but Src knockdown or activation-blocked groups showed minor lipid accumulation than the NC or PP3 groups (Figure 5(e)). Furthermore, knocking down the expression or blocking the activation of Src relieved the oxLDL-induced loss of VSMC contractile markers and the acquisition of foam cell phenotype (Figures 5(f) and 5(g)).

### 3.5. Tlr4 Mediated Sirt1/Sirt3 Alteration Which Regulated oxLDL-Induced Oxidative Stress and Foam Cell Formation in VSMCs

**Figure 6:**
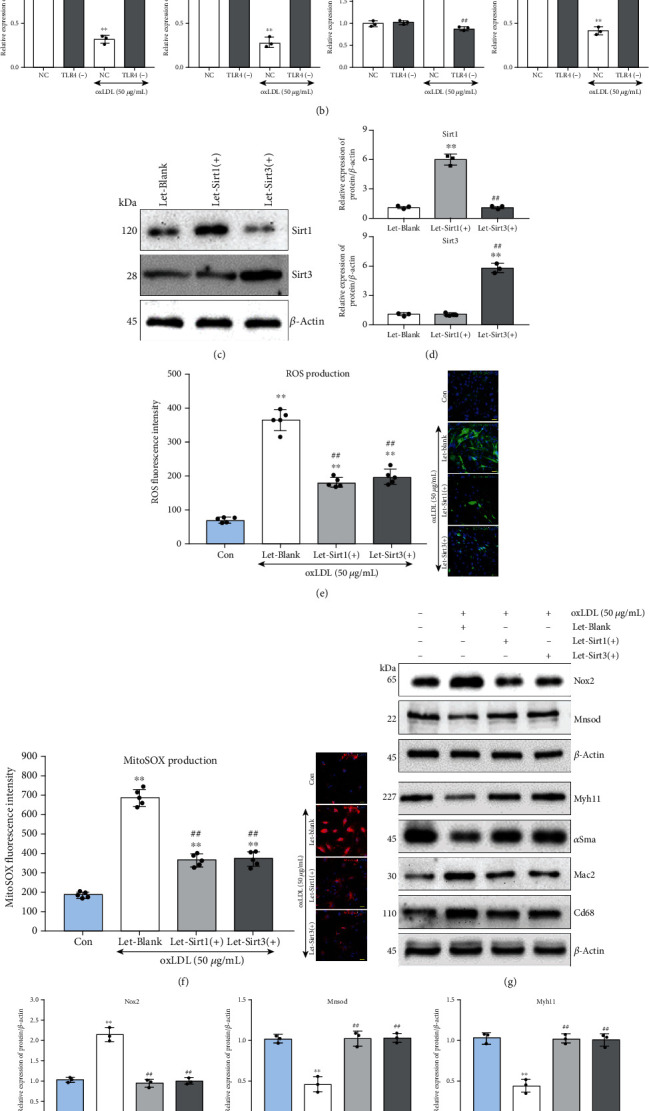
Overexpression of Sirt1 or Sirt3 inhibited oxLDL-induced ROS accumulation and altered cellular phenotype in VSMCs. All the western blot results were calculated using grayscale value, and *β*-actin served as an internal reference gene to normalize protein expression. (a, b) Tlr4(-) or NC VSMCs were treated with or without oxLDL (50 *μ*g/mL) for 48 hours; western blot was conducted to measure Sirt1, Sirt3, Nox2, and Monsod. (*n* = 3 per group, results were expressed as mean ± SD; ^∗∗^*P* < 0.01, as compared with oxLDL-untreated NC group; ^##^*P* < 0.01, as compared with oxLDL-treated NC group.) The recombinant lentivirus of Sirt1 or Sirt3 has infected into VSMCs for Sirt1 and Sirt3 overexpression. The VSMCs were treated with or without 50 *μ*g/mL oxLDL for 48 hours. (c, d) The VSMCs were infected by recombinant lentivirus of Sirt1 [Let-Sirt1(+)] or Sirt3 [Let-Sirt3(+)], respectively, for 72 hours; afterwards, the overexpression efficiency was detected. (*n* = 3 per group, results were expressed as mean ± SD; ^∗∗^*P* < 0.01, as compared with the Let-Blank group; ^##^*P* < 0.01, as compared with Let-Sirt1(+) group.) (e) ROS (DCFH-DA, green) and (f) mitochondrial superoxide (MitoSOX, red) were measured. (*n* = 5 per group, results were expressed as mean ± SD; ^∗∗^*P* < 0.01, as compared with the control group (untreated group); ^##^*P* < 0.01, as compared with Let-Blank group). (g, h) Western blot was conducted to measure the Nox2 and Mnsod (upper), as well as the VSMC contractile phenotype markers (Myh11 and *α*Sma) and foam cell markers (Mac2 and Cd68) (bottom). (*n* = 3 per group, results were expressed as mean ± SD; ^∗^*P* < 0.05, ^∗∗^*P* < 0.01, as compared with control group (untreated group); ^#^*P* < 0.05, ^##^*P* < 0.01, as compared with Let-Blank group).

Interestingly, we also observed that when Tlr4 in VSMCs was knocked down, the expression of Sirt1 and Sirt3 restored compared with the NC group after 48-hour oxLDL treatment accompanied by reduced Nox2 and elevated Mnsod expression, implying that Tlr4 contributed to Sirt1 and Sirt3 downregulation under oxLDL stimulation (Figures 6(a) and 6(b)).

To investigate whether Sirt1 and Sirt3 could antagonize oxLDL-induced ROS accumulation, mitochondrial superoxide, and foam cell formation in VSMCs, recombined lentivirus of Sirt1 [Let-Sirt1(+)] or Sirt3 [Let-Sirt3(+)] overexpression was transfected SMCs. The Let-Sirt1(+) or Let-Sirt3(+) significantly increased the expression of Sirt1 and Sirt3, respectively, without mutual interference (Figures 6(c) and 6(d)). Compared with the untreated group, the addition of oxLDL caused ROS and mitochondrial superoxide accumulation in VSMCs. However, Sirt1 and Sirt3 overexpression groups displayed alleviated oxidative stress (Figures 6(e) and 6(f)). Additionally, though oxLDL led to significantly elevated Nox2 and decreased Mnsod, Sirt1 or Sirt3 overexpression almost reversed such impact on these genes (upper, Figures 6(g) and 6(h)). Moreover, increased expression of Sirt1 or Sirt3 also relives the oxLDL-induced VSMC contractile phenotype marker (Myh11 and *α*Sma) loss and foam cell marker (Mac2 and Cd68) acquisition, which implied that Sirt1 and Sirt3 partly assist in VSMCs phenotype rebalancing under oxLDL stimulation (bottom, Figures 6(g) and 6(h)).

## 4. Discussion

In this study, we demonstrated that oxLDL induced the transition of VSMCs to foam cells by promoting lipid accumulation and ROS production via raising the expression of a key linking molecule-Tlr4. Furthermore, oxLDL-induced lipid and ROS accumulation and phenotype alteration in VSMCs were at least partly attributed to Tlr4-regulated Src activation and Sirt1 and Sirt3 upregulation. Our findings unravelled a crucial role of Tlr4 in oxLDL-induced foam cell formation in VSMCs.

The toll-like receptor family constitutes important members of pattern recognition receptors (PRRs), which identify particular ligands of receptor to evoke pathogen-associated molecular patterns (PAMPs) [21–23]. These innate immune responses were commonly activated in innate immune cells. Likewise, as a chronic inflammation process, atherosclerosis was closely bound with the continuous innate immune response triggered by activation of PPRs [24]. Basically, oxLDL is a principal component of endogenous lipid ligand that causes endothelial cell injury, accumulates in macrophage and VSMCs, and induces a cellular inflammation response [25, 26]. Tlrs were reported to participate in oxLDL-induced inflammation response [27–29], but an integrated expression feature of the Tlr family in VSMCs under oxLDL stimulation remained unelaborated. In our present study, we found that oxLDL raised the expression of Tlr4 more significantly, which was over 1.5-fold than other Tlrs in VSMCs. Such observations suggested that among the Tlr family, Tlr4 might serve as a major participant in oxLDL-induced alterations of VSMCs.

**Figure 7:**
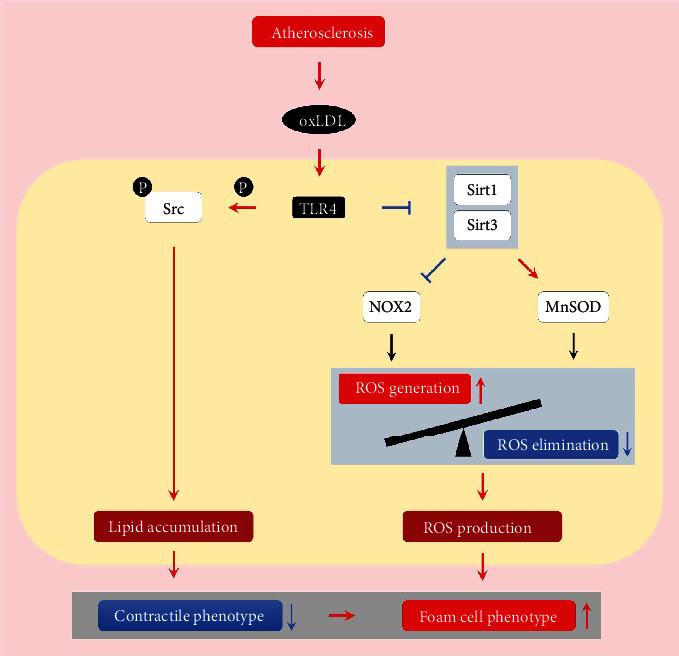
Overall network of Tlr4 in regulating oxLDL-induced foam cell formation in VSMCs.

Foam cell phenotype and inflammation response are two significant interrelated alterations in VSMCs that exacerbated atherosclerosis progression [6]. Our work provided evidence for the role of Tlr4 throughout the oxLDL-induced change in VSMCs. Previously, we certificated that Tlr4 was a crucial inflammation regulator in oxLDL-induced inflammatory cytokine expression/secretion as well as p38 and NF*κ*B activation [16]. Herein, we also detected the role of Tlr4 in foam cell formation in the VSMCs. In this study, besides gradient upregulation of Tlr4, we evidenced that knocking down Tlr4 could reverse oxLDL-induced phenotype change, which was characterized by emerging foam cell phenotype and weakened contractile phenotype in VSMCs. Taken together, we found that Tlr4 not only played a crucial role in the oxLDL-induced inflammatory response but also mediated oxLDL-induced foam cell formation in VSMCs (Figure 7).

Foam cell formation, characterized by the accumulation of intracellular lipids and the occurrence of inflammatory phenotype, is a hallmark in atherosclerosis progression [30]. Lipid loading in VSMCs activated multiple proinflammatory genes, suppressed expression of VSMC marker genes, and led to phenotype switching as well as inflammatory cytokine secretion [31]. Herein, we found that oxLDL evoked lipid accumulation in VSMCs, and knocking down the Tlr4 inhibited such lipid uptake in VSMCs, implying that Tlr4 mediated oxLDL-induced lipid accumulation and promoted subsequent foam cell formation. In a previous study, we demonstrated that Tlr4 directly participated in oxLDL-induced lipid uptake in macrophages by regulating Src kinase [17], indicating that Tlr4 was not only an innate immune receptor in inflammatory response but also a mediator for lipid accumulation. Since little was known about the role of Tlr4 in lipid accumulation of VSMCs, herein, we showed that Tlr4-Src signalling contributed to lipid accumulation of VSMCs and further demonstrated that the foam cell phenotype could be partly reversed after interfering Tlr4 or Src. These observations suggest that the TLR4-Src pathway might be a common regulation mechanism of oxLDL-induced lipid accumulation and foam cell formation in atherosclerosis (Figure 7). Actually, Src might be reasonable to serve as a downstream molecule contributing to the lipid loading in VSMCs. Firstly, the activation of Src may mediate the rearrangement of the cytoskeleton, which is a significant cellular alteration underlying endocytosis [32]. Additionally, Src signalling activates c-Jun N-terminal kinase and enhances the transactivity of c-Jun in response to LPS, thus triggering the expression of inflammation markers [33, 34]. Therefore, Src is likely to alter the lipid accumulation and inflammation response in the VSMCs.

ROS was a well-known mediator that exerts severe intracellular oxidative stress and prompts an inflammatory response, structural reorganization, and even cell phenotype transition [35, 36]. In our present study, we found that oxLDL promoted intracellular ROS and mitochondrial superoxide accumulation. In terms of this remarkable cytological event, we explored the remarkable cytological event from dialectical aspects, including both the ROS generation through Nox2 and Noxr4 and the ROS elimination via Mnsod, which constitute the balance of “in” and “out” on a “tank.” We found that oxLDL led to the overflow of ROS production through accelerating speed of “in” (increasing Nox2 expression but not Nox4) and decelerating speed of “out” (decreasing Mnsod expression) in VSMCs. Along with the reversed phenotype alteration of VSMCs, knockdown of Tlr4 also inhibited ROS and mitochondrial superoxide production, reduced Nox2 expression, and increased Mnsod expression, suggesting that the ROS production speed of “in” or “out” was regulated by Tlr4. These results indicated that Tlr4 mediated oxLDL-induced ROS accumulation through regulating the balance of ROS homeostasis, which might be another potential mechanism of Tlr4 during foam cell formation in VSMCs.

Since previous studies showed that sirtuin family members were located in nuclear and mitochondrial organelles, maintaining redox homeostasis via regulating the oxidative stress-associated genes [19], we also examined whether the sirtuin family participates in the Tlr4-mediated oxidative alteration during foam cell formation of VSMCs. We found that amid the sirtuin family, Sirt1 and Sirt3 were downregulated most significantly after oxLDL stimulation. As previous publications revealed that Sirt1 and Sirt3 were capable of suppressing ROS accumulation through inhibiting the activities of Nox and activating the Mnsod in ageing and carcinogenesis [37–39], we hypothesized that Sirt1 and Sirt3 might also serve as upstream molecules to regulate Nox2 and Mnsod expression in VSMCs during atherosclerosis. In line with these findings, we observed that overexpression of Sirt1 and Sirt3 upregulated Mnsod but downregulated Nox2 under oxLDL treatment. Furthermore, raising the expression of Sirt1 and Sirt3 inhibited the production of ROS and foam cell phenotype in VSMCs. These results illustrated that Sirt1 and Sirt3 participated in ROS accumulation and foam cell formation via regulating Nox2 and Mnsod expression, and regulating Sirt1 and Sirt3 might alleviate the oxidative stress and foam-cell formation in VSMCs. More importantly, when considering the reversed expression of Sirt1 and Sirt3 after knockdown of Tlr4, we concluded that oxLDL promoted ROS accumulation via the Tlr4-Sirt1/3 signalling pathway, thus inducing the foam-cell phenotype of VSMCs.

Our study demonstrated that Tlr4 is a critical regulator in oxLDL-induced foam cell formation of VSMCs via mediating Src kinase as well as Sirt1 and Sirt3. Beyond the role of Tlr4 in the inflammation response of VSMCs, we added a more integrated mechanism about TLR4 in VSMC phenotype transition under oxLDL stimulation.

## Figures and Tables

**Table 1:** The primer has been used for real-time PCR.

Gene	Forward primer	Reverse primer	Products size (bP)
Myh11	5′-AAG CTG CGG CTA GAG GTC A-3′	5′-CCC TCC CTT TGA TGG CTG AG-3′	238
*α*Sma	5′-GTC CCA GAC ATC AGG GAG TAA-3′	5′-TCG GAT ACT TCA GCG TCA GGA-3′	102
Mac2	5′-AGG AGA GGG AAT GAT GTT GCC-3′	5′-GGT TTG CCA CTC TCA AAG GG-3′	143
Cd68	5′-TTG GGA ACT ACA CAC GTG GGC-3′	5′-CGG ATT TGA ATT TGG GCT TG-3′	67
Tlr1	5′-CAA TGT GGA AAC AAC GTG GA-3′	5′-TGT AAC TTT GGG GGA AGC TG-3′	200
Tlr2	5′-AAG AGG AAG CCC AAG AAA GC-3′	5′-CGA TGG AAT CGA TGA TGT TG-3′	199
Tlr3	5′-CAC AGG CTG AGC AGT TTG AA-3′	5′-TTT CGG CTT CTT TTG ATG CT-3′	190
Tlr4	5′-ACC TGG CTG GTT TAC ACG TC-3′	5′-CTG CCA GAG ACA TTG CAG AA-3′	201
Tlr5	5′-AAG TTC CGG GGA ATC TGT TT-3′	5′-GCA TAG CCT GAG CCT GTT TC-3′	201
Tlr6	5′-TTC CCA ATA CCA CCG TTC TC-3′	5′-CTA TGT GCT GGA GGG TCA CA-3′	201
Tlr7	5′-AAT CCA CAG GCT CAC CCA TA-3′	5′-CAG GTA CCA AGG GAT GTC CT-3′	142
Tlr8	5′-GAC ATG GCC CCT AAT TTC CT-3′	5′-GAC CCA GAA GTC CTC ATG GA-3′	195
Tlr9	5′-ACT GAG CAC CCC TGC TTC TA-3′	5′-AGA TTA GTC AGC GGC AGG AA-3′	198
Tlr11	5′-CCA GGA CTG CAC CTT TTG G-3′	5′-GTG ACA CTG GTT GTA CGC AAT-3′	185
Tlr12	5′-TTG GAA GTT GTA CCT CGG ACT-3′	5′-GAA GTT GGG TAA GGT GCA GAC-3′	130
Tlr13	5′-GTT GTA ACC TGG ATG CCT AAG AC-3′	5′-GGC CTC TGT CAA GTT GGT GA-3′	198
*β*-Actin	5′-GAC AGG ATG CAG AAG GAG A-3′	5′-CCA CAT CTG CTG GAA GGT GG-3′	138

## Data Availability

Supporting data of this study are available from the corresponding authors on request.
